# Extracts

**Published:** 1883-09

**Authors:** 


### Extracts.

---

Overwork.—So much has been written and said about overwork, especially among Americans, where the tendency to overwork is said to be greatest, that very inaccurate notions prevail as to what constitutes overwork, and where the line should be drawn between healthful exercise of the functions, mental, physical and their abuse. The following timely and sensible remarks of *The Lancet* very clearly define this important distinction. The theory advanced will no doubt astound the growing class of American dilletanti, who nourished on the cant of the day, have taken refuge from the supposed evils of overwork in an apathic indifference, and go drifting along life's currents, deadening their energies and blunting their sensibilities from simple disuse of faculties which can never get beyond the germ stage unless developed by earnest labor.

In applying its comments on overwork to education, *The Lancet*, with no uncertain voice, speaks as follows:

A great deal has been said and written of late on the subject of "overwork," more particularly in connection with education. It is time that this question received the full elucidation it requires. It is natural that such a term as "overwork" should come into use, and that it should be popularly applied to all forms or classes of injury sustained by the organ of mind in the course of exercise, whether in result of excess or misdirection of activity; but it must be obvious to everyone possessed of even the most rudimentary acquaintance with physiology that the indiscriminate use made of this term is erroneous and misleading, and that the very hypothesis of *overwork* is in itself open to serious question, and what seem to be grave objections.

Let us recall to recollection for a moment what takes place in the case of muscular tissue under ordinary conditions of exercise, when intentionally developed by exercise and when overstrained by excessive exertion, or, in other words, "overworked." Moderate exercise, as we know, simply consumes the force generated, or, in more technical language, converts potential into kinetic energy. There is just as much material recuperation as will suffice to replace the elements utilized. Pushed a little beyond this as in "training" judiciously conducted, muscular tissue is first incited to a gradual increase in the amount of work it performs, with the effect of stimulating the recuperative faculty to a little more than merely compensatory energy, so that there is enough feeding to suffice for *growth* as well as *restitution*, and the muscle increases in bulk. This is not, as we know, due to any augmentation in the number of the elements composing the tissue, but to their increased development. They are not more numerous but they are larger. In point of fact the fibres attain greater bulk in consequence of the increased stimulation and the larger amount of food assimilated by the tissue, the supply of nutrient fluid, i. e., the blood, being augmented quite as much by the local demand for food as by any other condition which may be supposed to determine a special flow of blood to the part. If muscular exercise be increased too rapidly, faster than the rate of growth of the tissue, or if it be increased after the tissue has attained the full limits of a normal growth—which growth, it will be remembered, is simply compensatory to the work done, as in hypertrophy of the left ventricle of the heart,—there will be exhaustion: that is to say, an uncompensated consumption of tissue, and, if the work be further increased, exhaustion may proceed so far as to enfeeble the faculty of recuperation itself, to such an extent that it will no longer even replace normal waste. The stress of the work falls on the nerve centres, which of course are the sources of energy, but it seems probable that the terminal plates in the muscular tissue, which are probably reservoirs of nerve-force kept charged—as Leyden jars may be charged with electricity—for the purpose of local or reflex activities, may themselves be specially exhausted and rendered incapable of normal action, so that "cramps," stiffness—which is *rigor*, doubtless due to commencing coagulation of the myosin,—and local pain or tenderness often accompanied by fibrillar pains and twichings, due to local excitations, will ensue. At the same time the nervous system of the muscular apparatus being exhausted, the vaso-motor function is impaired, the arterioles of the part affected lose their tone, and the flow of blood through the region is retarded in speed, accumulation takes place, and what may be called atonic or passive hyperemia occurs, followed, it may be, by the phenomena of incipient inflammation; or, if there be not enough sf energy for that active state, then passive congestion, transudation, swelling, and œdema will supervene. This is a hasty and very general summary of what takes plece, or may occur, in muscular tissue when it is "overworked." Probably an analogous state of affairs supervenes in the case of nerve-tissue, and notably of the brain, when unduly exercised. There is, however, this essential difference between brain and muscle—namely, that inasmuch as the former is the more delicately organized, and in a sense the most important—albeit it seems likely that nerve-tissue may be developed while muscular cannot be—it is specially protected, so that before "overwork" does serious mischief in the case of nerve-tissue, there are nearly always very distinct indications that the limits of healthy activity have been reached. Under only moderate evercise nerve-tissue does not, as a rule, develop rapidly, nor does it accumulate strength—that is, force held in reserve and available for action. If the brain is to grow—that is to say, grow complex structurally—it must feed freely, consuming more than sufficient to replace its waste. If it is to feed, it must *work*. There is no way of stimulating the *structural* growth of brain except by intellectual exercise. This is a point of fundamental importance, and it is of cardinal moment in regard to that form of development by training which we call "education." So true, so inexorable, is this law, that not even *general* stimulation by work will suffice. If any special faculty of what we call "mind"—that is, brain function—is to be cultivated, it must be called out by special training—namely, by work of the special nature it is desired to elicit. Eor example, there is no reason to suppose the faculty of learning languages can be developed by exercise in mathematics, or the converse. This is a matter not sufficiently well recognized. A "strong mind" is a well-grown brain, and "bias of mind" is a brain with some one or more of its parts specially developed. Of course heredity has much to do with the question of capability of development, because the young animal is produced "in the likeness" of its parent; but so far as we, as educators, are concerned, our training must be special if we desire to get special results. In the process of brain-growth, or, more accurately speaking, of brain development, the elements of which the organ is composed are exposed to precisely the same risks and contingencies as the elements of muscular tissue under varying degrees of pressure by work, and, *mutatis mutandis*, the physiological, running into pathological, effects of progressive increase of work are similar to those we have attempted to recall. The faculty of recuperation is in danger of being itself exhausted, and depression of nerve and force and atonic congestion supervene. A matter of moment to remember is that although the brain is structurally a packing together of centres, with afferent, efferent, and inter-communicating nerve-fibres, it is itself supplied with nerves, and subject to precisely the same conditions of coarse distusbance as other organs. Sometimes we forget this fact, as at others we forget, not so much that it is an organ, as that it is composed of nervous tissue.

The conclusions we deduce from these facts are as follows: "Overwork," properly so called, is not so likely to occur, or if it occur, to do mischief, as irregular or disorderly activity. If there be not sufficient time for recuperation in the course of work, exhaustion must take place. If the work done be of such a nature as to put an undue strain on any one faculty, harm may be done, although the brain as a whole may uot be severely taxed. If the sudply of brain food be insufficient to enable the recuperative faculty to compensate by food for consumption in use, tnere must be exhaustion. If work be exacted when any indication of exhaustion is present, it is impossible that injury shall not be inflicted. It follows that educators have special need of care to avaid engaging the brains of their pupils in work for more than very short periods, and to provide intervals during which there mdy be rest of the centres specially taxed. Much mvy be done by changing the kind of work frequently. We are of opinion that no growing child should be kept longer than half or at most three-quarters of an hour at one task, or even the same description of work. Again, the great centres of relation should not be overtaxed. Vision, hearing, the speech centre, and the centre specially concerned with written language, whether in writing or reading, should not be wearied. Brain weariness is the first indication of exhaustion. The faculty of "attention" is perhaps one of the most easy vulnerable of all the parts or properties of brain-function. It is the faculty which most readily becomes permanently enfeebled, and when weakened entails most trouble in adult life. In children it is difficult to catch and fix the attention. No effort should be spared to secure this fixity of thought; but in order to avoid weakening the power of "thinking"—as distinguished from "thought drifting"—the teacher should not strive, or desire, to hold the attention by any effort on *his* part longer than it is voluntary given by the child. The slightest indication of exhaustion should at once be met by a change of task. If these hints, general as they are, can be reduced to practice, we think there is little fear of "overwork" or harm from brain activity. Desultory and insufficient work is more to be feared by far than "overwork" becaase the brain, like every other part of the organism, grows as it feeds, and it can only feed as it works.

---

THE TREATMENT OF SPASMODIC DYSMENORRHŒA.—(*Jno. M. Keating, M.D., in Med. and Surg. Reporter.*)—I do not know of a more unsatisfactory case to have on hand than that of a young girl, unmarried, just at puberty, with this form of disturbence, or one who has had it for several years, and has had associated with it the distressing embarrassment caused by the slightest allusion to the subject of her ailment, an extreme sensitiveness of the nervous system, hysterical hyperæsthesia, both physical and moral, which is caused by the long suffering and wear and tear of the system. I am sure that most physicians have experienced at some time the great difficulties of such a case. The fond parent, in bringing her daughter to you, expects you to *re rail* the function which has jumped the track of painlessness and regularity, and yet fondly hoping that it can be readily accomplished by a few doses of some magic drug, without the ordeal of the terrible examination which she has feared you will insist upon, and which has probably prevented the seeking of advice this long time. You know full well, as soon as you see the girl—the acne on her face, the muddy skin, or the chlorotic color, her languid air, or hysterical *nonchalance*, that further examination would not give you additional light. You hear the usual story of malaise headachi, perverted appetite, the history of cramp, of scanty flow at the onset, irregularity in appearance of the period, and total disregard to the thorough emptying of the bowels. The mother will tell you in confidence that the pain at the period is only cured by a little gin or brandy, and your own experience shows you that the amount of such must necessarily be increased at each time, in order to completely dull the increasing sensitiveness of the nervous system.

Probably an examination would only reveal to you a remarkable accumulation within the lower bowel, the discovery of which could have been arrived at by logical deduction prior to any investigation. When a person has been taking brandy or gin for any length of time for this form of dysmenorrhœa, you may be assured that constipation exists.

The mother tells you that she endeavors to make her daughter prudent, but that she is irritable, unlike herself as of old, and the daughter most naturally dreads your inquisitiveness, and seeks to keep back by monosyllable answers the true history of herself. The mother states that she "fears she will have spasms." Yes, they do use hot baths, ginger tea, hot vinegar, and finally the excited nervous system yields to free libations of hot gin and water, or a "little of the best brandy," and absolutely drugged, she will feel no pain or nausea, and vomiting will end the cramp.

Do most physicians know that the prescription of morphia and chloral, of "bromidia," of chloroform mixture, which they write at the bedside of the girl writhing in dysmenorrhœa from cold, etc., is renewed and renewed, is copied and carried from this place to that, is sent to friends, is recommended for sleeplessness, for neuralgic headaches, for nervousness, and for all the ills that flesh is heir to? The frightful proportions to which the habit of self dosing at each menstrual period has assumed, the fearlessness and frequency with which the hypodermic syringe is used, or the opium suppositories, shows that the habit is growing upon the community in the better classes, and that it needs at once most decided disapproval of the medical profession, who unfortunately are, in many cases, the innocent causes of the distressing evil, by thoughtlessly placing the weapon, without caution, in the hands of those who will abuse it.

In the treatment of these cases I have lately adopted a plan that has so far met with success, and I wish some of my medical friends who have the opportunity to do so would make the trial also.

First of all, relieve the bowel of its contents, give a large dose of castor oil, and see that the lower bowel is well empted by an enema. By the use of enemata you have a gowerful way of treating your case, and at the same time one whioh will not be abused by your patient. Just before the period is expected the patient should remain in bed with the hips well elevated and lying on her left side; her mother, nurse or some friend who possesses her confidence should administer an enema of at least a quart of thin strained oatmeal gruel, or if you prefer it the ordinary castile soap and water enema with some castor oil. This will empty the bowel. I do not recommend the fountain syringe, because I would prefer that the patient should have the enema administered to her, and not get into the habit of using it herself. Now, if the period should come on and be extremely painful, the anodyne can be administered by enema; of course a small quantity should be injected, as you wish it retained, and in conjunction with the hot foot bath, warm poultices to the abdomen, and hot drinks of some harmless tea, the relief will be soon obtained.

During the time intervening between the periods, I give the following:

℞.—Pul. ferri exsiccat..................
Potass. carb.........................aa gr j.
Ext. belladonnæ...................gr. 1-24.
Ext. aloes, soc.....................gr. ½.
Ext. gent. comp....................gr. j.
M.—Ft. pil. one. One after each meal, and also
℞.—Acid phos. dil.......................30 drops,
in water before meals. t. d.

I have found the *fluid extract of gelseminum* in *three* or *four* drop doses, t. d., a very valuable drug in averting the attacks of neuralgia. It should be taken for at least *three* or *four* days before the period in very severe cases. The plan of treatment suggested by Dr. Weir-Mitchell is necessary for many very obstinate cases. The generous diet or enforced feeding, rest, massage, *absence from home,* and the controlling influences which are embodied in such a course, are often a necessity for complete cure. In ordinary cases the patient must drink at least a pint of milk twice daily, rise early enough in the morning to get a thorough sponging from head to foot, and take a short walk before breakfast. So far the treatment has been calculated to strengthen the system and regulate the digestive functions. I have incorporated with it the use of electricity. I am surprised to find the great difference of opinion in regard to the value of electricity amongst my medical friends. Some speak of it as useless, others as troublesome, others say that it is most valuable but that those who use it should be familiar with all the intricacies of the subject. My experience gives me great confidence in the value of properly applied electricity, and though I have given attention to the subject, and studied the valuable works of Bartholow, Beard, and Rockwell, and the interesting articles of my friend, W. R. D. Blackwood, I am convinced that in many cases excellent results will be obtained, without, of necessity, putting the physician to the trouble and expense of time necessary to the thorough understanding of the subject of electricity and its applications. Exactly how a dose of castor oil works I do not know, and why an interrupted current or application of *Static* electricity should be more useful in amenorrhœa, and why a constant current is more successful in allaying the pain of dysmenorrhœa, I am not prepared positively to say; nevertheless it is the case.

Now, in the subject before us, I have found, with many others, that the *constant* current is very valuable, and it can be used with very little trouble to the physician and no discomfort or embarrassment to the patient. I use a *Flemming* battery of *thirty* cells, which I keep in order so that I am *sure* when I use I get a current generated.

Strange to say, many cases of want of success owe this to the fact that no current of electricity passes at all. "Get your current," is the first on the list of instructions. About two weeks before the expected period I begin the systematic use, usually three applications a week, lasting about ten minutes. I do not apply the electrodes directly to the uterus *per vaginam*, but I find that by placing an ordinary sponge-covered *electrode* over the ovarian region, the anode, or positive pole, and the cathode, or negative pole, to the spine, and allow it to remain until it becomes uncomfortably warm to the patient, and then removing it for a moment, I get the best results. I place the cathode first in the dorsal region over the spinal processes and gradually move it down to the sacrum. The anode should rest longer over the left than over the right ovary. I find that *twenty* is usually sufficient; the number to use will depend on the patient, using that number that will allow her to keep the electrode on the skin for about one minute without moving it. By this means your patient will not hesitate to come regularly for treatment—so little exposure is necessary, so little trouble on her part is required; and, in fact, the treatment is as satisfactory to the doctor, as it will detain him but a short time. My experience has been that in this way from *six* to *eight* applications will enable a patient to pass comfortably over the following period. In some cases the result is astonishing, and in the meantime by strengthening the system, and inducing regular habits, a permanent cure will be obtained without the use of *anodynes* or *stimulants*. I have noticed that such cases, from school habits, have acquired the stooping posture, and, as I believe that this is a most potent cause of dysmenorrhœa, I have always endeavored to strengthen the muscles of the back by the use of the *interrupted* current to the muscles several times a week, and a system of gentle gymnastic exercises.

In summer, rowing, swimming and lawn tennis, will be most valuable when indulged in in moderation.

I do not mean to convey the impression that immediate cure will follow the relief obtained in such cases always. The treatment should be persisted in, perhaps reducing the number of applications before each period, until the patient's health and strength have so improved as to warrant the belief that the pain will not recur. The *electricity* takes the place of the *anodynes.*

One of my patients who had suffered agony month after month, and was only relieved by drinking hot gin and water in large quantities, after about *six* applications menstruated without the least *pain*, and the next period was passed in the same way; but the next one occurred after a fatiguing day of standing, nursing a sick mother, and the pain returned, and she was obliged to have recourse to the battery, which again relieved her. This case was one of remarkable improvement, and had withstood all kinds of medicinal treatment, and had been under observation for some years.

---

Recent Changes in the Method of Medical Instruction.—Dr. E. N. Whittier, of Boston, read a paper on this subject. A sketch of the old way of receiving a medical education was given, the two-years' tickets for four months' tuition and a certificate being formerly all that was required. The value of the certificate could be seen when it was shown that the preceptor very seldom, if ever, asked his pupil a question concerning medicine, the student's time being spent in preparing drugs and in looking after the chores; and in the meantime, if a spare opportunity presented itself, he had the use of the doctor's books. This condition the speaker contrasted with the method of the present day, when four years' continuous work was expected, with, in many cases, an additional year in a hospital.

There was one faculty that was formerly trained which was now lost sight of in the anxiety to trace the science of medicine, to-wit, individual observation. Dr. Jackson, in speaking of Dr. Holyoke, the first president of the Massachusetts Medical Society, said: "After a time he allowed me to walk with him," and this stimulation of the powers of observation was the great strength of the old system.

The object of the leadlng schools was not to graduate large classes, but to graduate well-qualified classes. In addition to extending the course to four years, as Harvard had done, it had been proposed to include in the preliminary examinations anatomy, chemistry, and physiology, so that laboratory investigation might be begun at once; and in the second year to have didactic instruction to give place to clinical investigation. The art of observation must be cultivated, and the best way to accomplish this was to divide the classes into small sections, so that each pupil was brought into personal contact with the instructor.

The treatment in every case should be carefully marked out. If this were not done, but simply the history of the disease given, he who taught the art of healing was not a physician, but a naturalist.

No class exercise was complete unless the blackboard was used, on which a display of the rational and physical signs was made, and a classification of symptoms given. The true secret in medical teaching was for the instructor to carefully do his work the first part of the year; to train the students in the correct way to investigate a case, and have each pupil do the work before the class the latter part of the year.

---

A SHORT time since Professor Joylet, of Bordeaux, nearly lost his life in endeavoring to demonstrate, by Grehaut's method of inspiring hydrogen, the lung capacity to his pupils. (*British Medical Journal*). He had prepared the hydrogen gas, but, wanting some acid, he sent for it to a neighboring laboratory, poured some into the apparatus, and then made the inspirations necessary for the demonstration. The acid he had used, though sold as pure, contained arsenic, so that, instead of pure hydrogen, M. Joylet had inspired arsenuretted hydrogen. Notwithstanding sudden feelings of illness, he had the great courage to continue his lecture to the end, but was obliged to go home immediately, overcome by a fearful attack of headache, vertigo, and symptoms of syncope. Still more serious symptoms supervened, which caused great alarm, and during some days M. Joylet wss very ill. Fortunately, there were no serious results, and although still very weak,

M. Joylet is, to the great joy of his pupils, quite out of danger.

---

Good Remedies Out of Fashion.—In an address on this subject, delivered at the annual meeting of the Metropolitan Counties Branch of the British Medical Association, by the president, Dr. C. J. Hare, late physician to University College Hospital, the lecturer made a strong plea for return to the use of certain measures, notably emetics and bleeding, which he admitted were formerly, perhaps, abused, but which he considered in their appropriate cases invaluable. He alludes to practitioners who have never used either of these agents, and proceeds to cite cases from actual experience where such measures are necessary. We quote two from the *British Medical Journal:*

"In suffocative bronchitis the effect of emetics is sometimes magical, and by their administtration in such cases not only is immense relief given, but I verily believe—I am certain—that lives are saved. You are called to a patient who has been ill a few days, with increasing dyspnœa; she is sitting up in bed (I draw from nature), for to lie down is impossible; she is restless and tossing about; the lips, and indeed the whole face, blue; the eyes watery and staring; the pulse quick and small; the cough constant; the expectoration semi-transparent and tenacious; over every square inch of the chest, front and back, from apex to base, you find abundance of rhonchi; moist, sonorous, and sibilant ones in the upper part of the lungs, and muco crepitant or mucous rales toward the base. Ammonia and stimulants, right and good in their way, perhaps, in such a case are too slow in their action; the patient is, in fact more or less slowly, more or less rapidly, suffocating. An emetic of twenty-two grains of ipecacuanha in an ounce of water is given; in ten or fifteen minutes the patient vomits, and brings up a huge quantity of that tenacious mucus, and the whole aspect of the case is altered; the distressed countenance is relieved; the breathing is at once quieter; and the patient is able for the first time for the past twenty-four hours to lie moderately low in bed, and to get some sweet, refreshing sleep.

The patient is, in fact, rescued from the extremest peril, and in this case, and in many similar ones too, I believe, from otherwise most certain death. Of course, in such cases the emetic is not given for its effect on the stomach, but for its collateral effect in mechanically clearing out the enormous amount of secretion which accumulates in the bronchial tubes, and which the patient is otherwise quite incapable of getting quit of; and thus the half-choking, almost asphyxiated, condition is changed for one of comparative comfort, and time is gained for the action of other appropriate remedies. No doubt the secretion may, and often will. accumulate again; and I have not hesitated again in bad cases to repeat the same good remedy; but it is a fact, and a very positive one too, that, quite contrary to what those who have had no experience in the plan suppose, the system rallies instead of being depressed under the action of the remedy.

"There is a class of cases in which the right heart is engorged with blood, and in which the only hope of rescuing the patient from death is by bleeding. A man of middle age (I again draw from nature) has considerable chronic bronchitis, with some congestion of the lungs, and, like many other unwise persons, he goes to a southern watering place instead of remaining in his room and in a uniform temperature. Becoming worse, he determines to return home, and travels on a cold spring day; his dyspnœa is so much worse on the journey that his friend and the fellow-passengers doubt whether he will arrive home alive; and when his carriage meets him it is with the greatest difficulty he is conveyed to his house and got into his drawing room. You are at once sent for, the message being that the patient is dying, and when you arrive you find that this is the fact. He is sitting in a chair (to lie down is impossible for him), his face is blue and swollen, his lips purple, the eyes suffused and staring, his heavy, gasping breathing you have only too distinctly heard and recognized as you ascended the stairs, and when you see him you find his chest heaving, and each short gasping inspiration followed by a long wheezing and moaning expiration; his lungs are full of moist sonorous, and mucous and submucous, rhonchi, and scarcely a trace of vesicular respiration is to be heard, and he is pulseless. He looks to you beseechingly, and gasps out, in scarcely articulate words, that he is dying. This is but too true. Now, the treatment for such a condition at the present day is "to pour in stimulants" (though the patient can scarcely swallow). Brandy and water are given, and ammonia, and perhaps ether; then, if the patient live long enough to have them made, mustard poultices are applied to the chest, and to the calves, and to the feet, and the patient is fanned, and the patient dies. Something has been done, but that which true pathology—and, indeed, common sense, unshackled by prejudice, custom and fashion—would dictate, has been left undone. Appearances have been saved, but not the patient's life.

"The fact is, that here the danger lay in the right side of the heart being gorged with blood, so that it was impossible for its stretched and distended walls to contract and and to propel forwards the thick and blackened blood. Relieve that poor oppressed, distended heart, and all may be well! Open one of those veins which are, with every systole of the heart, tending to carry more and more blood to this already distended right ventricle, and all may yet be well with your patient. Sometimes this blood-letting, in extreme cases, is no easy matter; it may be necessary, before you can effectually open the vein, to place the patient's arm in warm water, so as sufficiently to distend the vein, and even when the ligature has been efficiently applied, and the vein well opened, you may have to press and squeeze and rub upwards the arm before a drop of the thick and tarry blood will flow. But, when it *does* flow at length freely, oh, what a marvelous change may you see take place!—the breathing becomes quieter, deeper, and less noisy, the haggard face resumes the appearance of tranquility, the blueness of the skin is replaced by a more natural tint, the pulse becomes more and more distinct, and, in a word, the choked-up heart is set free. This is no fancy picture. Every word is simple truth, and I appeal for confirmation to the memory of every senior member present who recollects the experience of his earlier days, and who can also probably tell you that the after-progress of such cases was sometimes almost miraculously rapid, so that in a few days even the patient might become convalescent."

---

WOMAN AND HER AILMENTS.—(*Jane M. Baker, M.D., in Medical Truth*).—Let a mother bear in mind that her first duty is to develop the physical organization of the child; for how can we expect a sound mind to dwell in a weakly body? Therefore the child must grow and gain in strength. Good nourishment which the plainest food imparts, must be given to the child at regular hours. Sufficient sleep and exercise must help to digest the food. The younger the children are, the more sleep they require.

Some mothers treat their little girls as if they were little dolls; they want them to look "so nice," and are trying to teach a little three-year-old girl to behave like a lady.

Such nonsensical mothers ought not to have any children. Comfort is what they ought to have; if they romp about and are wild, so much the better for them, and what of it, if they get a little dirty, there is plenty of water to wash them clean.

If mothers would exact strict obedience of their children, providing for their wants, contributing to their amusement, without yielding to their whims; they would add greatly to their physical welfare as well as to their moral training.

A very important question in regard to the health of children is when they should go to school.

There are laws in certain countries that when a child is six years old it *must* go to school. I believe in compulsory education; but, to carry out a law like this strictly to the letter is cruelty in some cases.

If the child is well developed and healthy, then let it go to school; but not otherwise. If they have to wait with their a, b, cees until they are seven or eight years old, they have not lost anything, if their health has thereby gained.

And now let us skip over a few years, and see a young miss of fifteen. Let me describe to you not an imaginary case, but one taken from real life.

Miss R. is very near sixteen. She is tall, slender, poorly developed, pale, with bright, intelligent eyes. She has not menstruated yet. Let me describe to you one day of her life, which is a type of all the rest. She rises at eight, unrefreshed and languid. Why so the sequel will show. She has no appetite, so a cup of tea is taken and a piece of toast is worried down. She is in a hurry, for at nine o'clock she has to be at school, a great distance off. She gathers up a big pile of books. What are they? English, Spanish, French, Latin, (O horrors!) Greek, Logic, Natural Philosophy, Algebra, Conic Sections, Astronomy, etc. A cold lunch goes into her satchel. She is ambitious, and studies hard, acquitting herself well at recitations. At four she is at home, and her music teacher is waiting, who is very particular that she should sit straight on the stool. After an hour of hard work she retires to her room. Oh, how much she would like to lay down and rest; but she must dress for dinner at six. This over, she must go to the parlor and often entertain the family or guests at the piano.

Eight or nine o'clock finds her in her room writing compositions, solving most difficult problems in mathematics, and trying to define the writings of the classics of Rome and Greece. Midnight still sees her at her labor. At last, weary to death, the brain all muddled with a chaos of abstract problems, she retires to sleep. To sleep? It is more a comatose condition than a sleep. And the silly mother wonders what makes her girl look so delicate. Why has she not menstruated yet? She had when she was fourteen—she was brought up in the country, a plump, robust girl. Look once more at her daughter, with all her accomplishments—languages, mathematics, music, drawing. Mind, all this is in her brain and the tips of her fingers; the rest of her body is a wreck. Who wants her for a wife? If I was a young man, poor and without business, her large dowry might tempt me to take her. What does that girl need? A doctor? No. Medicine? "Throw physic to the dogs!" What, then? Common sense, and exercise on meadows and fields and in the woods.

Menstruation ought to appear at the fourteenth or fifteenth year in our climate. In southern climates it appears much earlier (a Hottentot girl may be a mother in her eighth year), and the farther north the later it appears. Thus, in Iceland, Norway and Sweden girls approach that period when they are nineteen or twenty. When it does not come at the proper time, the so-called emmenagogues are useless, even dangerous. But the rule is to build up the constitution by proportioning well mental and physical exercises and rest.

The only peculiar disease which occurs before puberty is inflammation and ulceration of the vulva. This may be overcome by a wash with borax, and cleanliness, and a mild laxative.

Children of full habit, with eruptions on their skin, must eat no eggs, have a plain food, and be kept very clean.

---

An Obstetrical Expedient--(*V. M. Richard, M. D., in Medical Times.*—There is an expedient which I have used with such great advantage that I wish to lay it before the profession, that some other may be relieved as signally as I have been. To illustrate, I will give a few cases:

Case 1.—Mrs. W., colored, æt. 35. In labor with eighth child. Always had easy labors. Membranes spontaneously ruptured several hours before my arrival. Pelvis large and roomy. Head rather small. Parts relaxed and os dilated. Pains good, but head would not engage properly. In the interim between two pains the head would go back, and could scarcely be felt After waiting several hours, I determined to take her up. Placed her on her knees by the bed, and in about fifteen minutes the child was born. No bad result.

Case 2.—Mrs. L., white, æt. 52. In labor with tenth child. Youngest seven years old. Dilation retarded, pains feeble. Gave chloral and quinine. After waiting several hours the head engaged, and all passed well until the vertex was about to sweep under the pubic arch. There it stuck. Extension could not take place, and the child could not be born. Her pains were now good, and an extensive *caput succedaneum* was forming. I waited till I feared for the life of the child, when I urged the foreceps. To this she strongly objected—absolutely refused. As a *dernier ressort*, I put her on her knees by the bed. The effect was perceived at once; the anterior part of the head went back against the perineum, and the occiput swept under the arch. The space gained could easily be recognized. The caput had previously covered all the part which could be detected by the finger, but now the cranium could be felt all around it. Extention took place, and she was speedily delivered.

Case 3.—Mrs. R., white, æt. 22. Second child. Membranes ruptured. Woman very stout, and abdominal tumor very large. Found head engaged, and fitting tightly. When on her back the fœtus would fall toward the spinal column; when on the side, it would fall laterally. Could not keep it in the axis of the pelvis, and her labor would not advance. Absolutely refused the foreceps. Put on her knees by the bedside. In this posture the fœtus kept its position, and, the uterine forceps being applied with advantage, delivery was speedily accomplished. The case was complicated with adherent placenta and hourglass-contraction; but, as she had suffered from this in her previous labor, it could not have been the result of the position.

These three cases illustrate several of what I consider the indications of the knee position in labor. All the writers on obstetrics speak of the advantage to be gained by sitting, standing, and walking around during the first stage of labor.

None, so far as I have seen, advise this. Atkinson says the position on the knees is used in Ireland. Engelmann has shown us that the almost universal primitive position is to have the body either completely or partially upright. While preparing this, I was told by an old professional friend that Meigs details several cases where he resorted to this position. In the systematic works of Ramsbotham, Leishman, Churchill, Playfair, Meigs and Lusk, I have found nothing except the horizontal position recommended, for the second stage of labor. In all my cases the dorsal and right and left lateral decubitus were successfully tried, and with no permanent results.

In none of my cases was delivery so rapidly accomplished as in the first. The child was born almost before I was aware of it, and that after I had waited for several hours with the woman in bed. Had the foreceps been used in this case it would not have been devoid of danger, as their passage into the uterus would have been necessary. In the second case the delay was due to the rigidity of the parts. The head could not pass sufficiently far back to allow the vertex to sweep under the pubic arch, and hence the delay. By changing the direction of the uterine action the necessary space was gained. With the foreceps the same result could have been accomplished more speedily, but she utterly refused them, though urged by myself, her husband and the nurse. In the third case the delay was due to the relaxed abdomen and the trouble in keeping the fœtus in the axis of the pelvis. When in the upright position there was no trouble of this kind, and her labor advanced to a speedy termination.

In these cases the placenta may be delivered while the woman is on her knees, or she may be put back to bed first. If there is no delay, the better plan is to remove it before putting her back, as by so doing a great deal of filth and dirt will be kept out of the bed. The woman will not be soiled, or compelled (as is so often the case) to lie in a pool of her own discharges. Indeed, one great advantage is the keeping of the bed clean. Some women will object to this position because they are too weak, others because it is indelicate ; but by stating the case clearly, and using some tact, the attendant ought to have no serious trouble in having his wishes carried out.

---

Reflex Irritation —(*Arthur L. Worden, M.D., in Iowa State Medical Reporter*).—Very many of the pains and ailments we have to meet with are reflex symptoms. A case was once brought for treatment, supposed to have hip joint disease (coxarum morbus.) Previous to this, however, the youth had had his knee entirely blistered, on the suppoition that he was threatened with inflammation of the knee joint. A careful examination led to the following prescription :

R.—Pulv. santonini........................gr. XII.
Hydrargyri chlorid mite............gr. V.
Pulv. rhei............................gr. XVI.

Mix et. ft. chart No. IV.

Sig.—One powder every second morning before breakfast.

During the next ten days the patient passed large quantities of *ascaris lumbricoides*, and all symptoms of the hip and knee trouble passed away.

The following case is almost unique: J. P., aged 13 years, came to my office April 21st, complaining of headache and a tender spot on the top of his head. I found a place about the size of a silver dollar that was extremely sensitive. Simply to brush the hair lightly with the hand would cause the patient to shrink. As the case had already been in the hands of several physicians and was considered chronic, I made no promises.

Questioning failed to throw any light on the case. I suspected some genital irritation, but patient had no bad habits and said he was not troubled any in regard to his sexual organs. Examination, however, revealed the fact that the prepuce was firmly adherent to the glans. My diagnosis was made. On 23d I "peeled off" the adherent prepuce—Dr. D. W. Smouse administering the anæsthetic. Absorbent cotton was placed between the denuded surfaces and they were allowed to granulate. In a few days they were entirely healed and in two weeks the soreness had entirely disappeared from the top of the head, the headache gone, and he was improved in all respects.

In May, 1880, Mrs. M. brought her son, aged four years, complaining of pain in the epigastric region. Examination revealed no symptoms that could be relied upon to make a diagnosis. I prescribed for dyspepsia and told them to return if he did not get better. June 10th they returned with the report that he was worse than ever. I stripped the child and examined him thoroughly. Found phymosis and diagnosed reflex irritation from adherent prepuce and deposit of calcarious matter about the corona glandis. I operated, under an anæsthetic, June 12th, and found as I had predicted. The patient made a fine recovery from the operation, and the pain in his epigastric region entirely disappeared.

At present I am treating a case of "weak legs" in a child, of nearly three years, in which I ascribe the whole difficulty to phymosis. I have circumcised him and expect a complete cure of the deformity.

---

TRICYCLES FOR PRACITIONERS.—I have recently been riding the "Omnicycle," with a view to test its capabilities as a tricycle for all round work, both in town and country. I consider that it is just the machine needed by the medical practitioner. It is a machine which is worked easier and with less fatigue than any yet in the market—as the crank and ordinary chains are dispensed with, and the rider's strength is directly applied to the segment of a circle on the main shaft. The peripheral surface can be made to expand or contract at the will of the rider, so that his force is directly communicated to the driving wheels with equal power throughout the whole of their revolution. All dead points are thus avoided, and an alteration of power is obtained without increase of friction or variation in the length of tread. The gear can be altered whilst riding, and three powers obtained: viz., 50-inch for the level, 40-inch for ordinary inclines, and 30-inch for stiff hills. The machine will mount hills (with comparative ease) which no bicyclist, and very few tricyclists, would care to mount. In fact, with tha "Omnicycle," hill-climbing, instead of being a toil, becomes somewhat of a pleasure. I have myself ridden the machine over some of the most hilly roads round Leeds, and up the steepest streets in the town. There is no fear of the machine running back on a steep hill, as it cannot do so; but there is a "reversing wheel" which will enable the machine to be rnn either backward or forward for stabling or other purposes. The tricycle only weighs about ninety-five pounds, but is made of the best workmanship, and will stand a good deal of wear. I know of no machine which will so perfectly answer the requirements of practitioners in hilly districts, and for town use. It is far superior to a horse, and may be ridden with much less fatigue. The motion is particularly easy. It is manufactured by Messrs. Singer & Co., of Coventry, a firm which turns out the best workmanship. I would advise any medical man who is thinking of a "mount" to see and try the "Omnicycle." H. A. ALBUTT, M. R. C. P., etc.

---

DEATH FROM THE STING OF A BEE.—" From time to time," says the *Lancet*, " we are startled by the news of a death following so closely on the sting of a bee that no reasonable doubt can be entertained of the causal relationship. The occurrence undoubtedly belongs to the chapter of accidents ; and an explanation can only be obtained by considering those kinds of things which are of an exceptional nature. A sting of an ordinary bee on an ordinary man is perhaps never followed by anything more than a local reaction. To explain the lethal effect, therefore, we must suppose that the virus of the bee was of an unusual nature, either as a result of admixture from without or as a consequence from some disordered action of the physiological processes of the bee. If the fault do not lie in the insect, then we must turn to the other factors of the resultant effect. There can be no doubt that the injection of the venom directly into a vein is a very dangerous matter ; and it is possible that this may be the accidental circumstance so necessary to afford a reasonable explanation. We learn from the *Sheffield and Rotherham Independent* that a small farmer, aged fifty-nine, while in good health and working in his garden, was stung in the eyelid by a bee ; the signs of collapse rapidly set in, and the unfortunate man died within half an hour of the receipt of the sting. It is worthy of remark that the daughter stated that her father had been twice previously bitten by a bee, and was very ill on each occasion."

---

ERGOT AS A PREVENTIVE OF THE AURAL TROUBLES OCCASIONED BY QUININE AND SALICYLATE OF SODA.—In three cases of acute articular rheumatism, Schilling has observed that the prolonged administration of salicylate of soda in doses of ℈vss–ʒijss a day, causes permanent aural trouble. The membrana tympani was thickened and of a cloudy aspect. A fourth case, which in two successive days had taken single doses of grs. xxx of sulphate of quinine, became subject to roaring in the ears and deafness. This is not an unfrequent effect of quinine or the salicylates administered in large doses, and Schilling proposes to administer ergot with these drugs in order to prevent this vascular paralysis; for example he gives the following:

Ergotine............................grs. xv.

Salicilate of soda......................ʒijss.

Water..................................℥vj.

S.—A large spoonful every hour. Of eighty-seven patients who have taken this prescription, three-fourths have had no aural symptoms, and the same was noticed in nine other patients who took quinine and ergotine in equal parts. This mixture will be equally preventive of the amblyopia which sometimes follows large doses of quinine. —*Progres Med.*

---

Detection of Stone in the Bladder of Children.—I wish to call the attention to the ease with which, in children, when thoroughly anæsthetised, a stone in the bladder may be detected, and its size and shape estimated, by a finger introduced into the rectum, and one or two fingers of the other hand placed on the abdominal wall; the stone being felt between them.

In my last two cases of lithotomy, I was able in this manner to judge the size of each stone to within a few grains; one, which I estimated at ten grains, proved to be twelve grains, and the other at twenty-five grains was thirty grains. In each case, I had previously found the stone by the sound.

Probably others have noticed this fact, but I have not seen it mentioned before.—*Thos. Sansome, M.R.C.S.*

---

Tuberculosis in Infants.—Dr. J. Lewis Smith, in *Med. Record*, thus speaks of his treatment of this disease:

Whenever, therefore, I am called to a young child with a chronic cough and wasting, I do not wait for a more accurate diagnosis, but if there be no diarrhœa to contra-indicate it, prescribe cod liver oil with the hypophosphites, frequently adding the syrup of the iodide of iron, since the strumous cachexia is so apt to be present with probably caseous substance, in some part. Such a case requires the utmost attention to the hygenic management, pure air, nutritious and easily digested diet, into which milk enters largely, the juice of meat or meat-broths, prepared at a temperature of 100°, so as not to coagulate the albumen. A favorite prescription in two of the asylums of this city for these infants with chronic cough and wasting, whether or not tuberculosis be diagnosticated, is the following, to be taken between the doses of cod-liver oil:

R.—Ammon. carbonat., ferri et ammon.
citrat............................aa. gr. xxiv.
Syrupi simplici....................℥ iij.

M. Dose, one teaspoonful every two or three hours to a child of one year.

---

Doctors.—The proportion of doctors to population is given as follows by the *Siglomedico:* France 2.91 per 10,000; Germany, 3.21 per 10,000; Austria, 3.41 per 10,000; England, 6 per 10,000; Hungary, 6.10 per 10,000; Switzerland, 7.06 per 10,000; United States, 16 24 per 10,000. As the mortality is greater per 1,000 in all the above countries than the United States, it would seem that the 1,500 turned out yearly are a benefit rather than a detriment, which refutes the assertion that we have too many medical colleges in this country. The more the better, and the more medical students graduated, if they are good ones, the better class of physicians we shall have to administer to the sick, for the *best* will remain in the profession while the poor must seek a livelihood somewhere else.

---

Poisoning From Eating Plum Stones.—Dr. Seferowitz (*Wein. Med. Blatter*) saw a case of hydrocyanic, or cynanogen, poisoning in a boy, twelve years of age, from eating the kernels of plum stones.

The symptoms were: convulsions, with loss of consciousness, which lasted for several hours, dilation of the pupils, tetanus and trismus, cyanosis and dyspnœa.

An emetic was given; the child was dipped into a warm bath and then treated with a cold shower, and artificial respiration was kept up. By these means, after twelve hours, consciousness returned. The patient was still very faint, and the pupils were dilated for many hours longer.

The boy stated that he had eaten a whole pocketful of plum stone kernels.

---

Castor Oil and Glycerine.—A mixture which is of an agreeable flavor and in which the nauseous smell of the oil is efficiently disguised, can be made thus:

℞.—Ol. ricini............................ʒ j.

Glycerini............................ʒ j.

Tr. aurantii............................mxx.

Tr. senegæ............................mv.

Aquæ, cinnam, ad............................℥ ss.

This forms a beautiful emulsion, is easily taken, even by children, and if administered at bedtime will produce a gentle motion the following morning.—*N. Y. Med. Journal.*

---

Transfusion of Pure Water.—Dr. Coates reports a case of transfusion of pure water, warmed to the proper degree. The patient was a primipara, twenty-seven years of age. The cause of collapse was an alarming hemorrhage on the ninth day after child-birth. Some twenty-two ounces of water were allowed to enter the median cephalic vein through a Jennings siphon. The result was striking, and convalescence speedy.—*London Lancet.*

